# Bioethical reflexivity and requirements of valid consent: conceptual tools

**DOI:** 10.1186/s12910-019-0385-7

**Published:** 2019-07-04

**Authors:** John Barugahare

**Affiliations:** Department of Philosophy, Makerere University, P. O. Box 7062, Kampala, Uganda

**Keywords:** Valid consent, Health-related research, Bioethical reflexivity, Research ethics, Ethical guidelines, Bioethical decision-making

## Abstract

**Background:**

Despite existing international, regional and national guidance on how to obtain valid consent to health-related research, valid consent remains both a practical and normative challenge. This challenge persists despite additional evidence-based guidance obtained through conceptual and empirical research in specific localities on the same subject. The purpose of this paper is to provide an account for why, despite this guidance, this challenge still persist and suggest conceptual resources that can help make sense of this problem and eventually mitigate it’.

**Main body:**

This paper argues that despite the existence of detailed official guidance and prior conceptual and empirical research on how to obtain valid consent, the question of ‘how to obtain and ascertain valid consent to participation in health-related research’ *cannot always* be fully answered by *exclusive* reference to pre-determined criteria/guidance provided by the guidelines and prior research’. To make intelligible why this is so and how this challenge could be allayed, the paper proposes six concepts. The first five of these are intended to account for the persistent *seeming inadequacies* of existing guidelines. These are *fact-skepticism; guideline insufficiency*; *generality; context-neutrality* and *presumptiveness*. As an outcome of these five, the paper analyzes and recommends a sixth, called *bioethical reflexivity.* Bioethical reflexivity is reckoned as a handy tool, skill, and attitude by which, in addition to guidance from context-specific research, the persisting challenges can be further eased.

**Conclusions:**

Existing ethical guidelines on how to obtain valid consent to health-related research are what they ought to be – general, presumptive and context-neutral. This explains their seeming inadequacies whenever they are being applied in concrete situations. Hence, the challenges being encountered while obtaining valid consent can be significantly eased if we appreciate the guidelines’ nature and what this means for their implementation. There is also a need to cultivate reflexive mindsets plus the relevant skills needed to judiciously close the unavoidable gaps between guidelines and their application in concrete cases. This equally applies to the gaps which cannot be filled by reference to additional guidance from prior conceptual and empirical research in specific contexts.

## Background

Despite existing international, regional and national guidance on how to obtain valid consent to health-related research, in practice valid consent remains both a practical and normative challenge. Unfortunately, this challenge still persists despite voluminous conceptual literature and empirical research conducted in specific localities that provide additional evidence-based guidance on how to obtain valid consent as cited elsewhere [1, 2]. This paper aims at 1) accounting for the persistence of *seeming inadequacy* of existing ethical guidelines despite additional guidance from both conceptual and empirical research intended to close the gap and, 2) make intelligible practical means by which to compensate for the persisting gaps. To accomplish these two objectives, drawing illustrations mainly from the *International Ethical Guidelines for Health-Research Involving Human Participants* provided by the Council for International Organizations of Medical Sciences (CIOMS) [3], this paper proposes and analyses six conceptual tools: *fact-skepticism; guideline-inadequacy*; *generality; context-neutrality; presumptiveness* and *bioethical reflexivity.*

Generally, ongoing discussions in research ethics particularly the topic of ‘valid consent’ for participating in health-related research are mainly concerned with the issue of the rigor of the process of obtaining consent. [4–9]. Concerns about the quality of informed consent have further deepened with a recent increase in the volume of genomics research in low resource settings [10–13]. Common questions shaping this discussion pertain to the type and amount of information study participants should be given [14, 15]; the best means to deliver such information [16–20]; the language of delivering such information and concerns about accuracy of translation of technical terms to enable sufficient comprehension [21–24]; the problem of vulnerability that usually conceals lack of freedom [25–28] among others. A growing body of work has been conducted recently in various local settings on these and related challenges and has produced additional and more localized evidence-based guidance on how valid consent ought to or could be obtained [2, 29–36]. However, regardless of all this guidance, obtaining and ascertaining valid consent remains both a normative and practical challenge in concrete situations. To facilitate an appreciation of the conceptual tools proposed in this paper, the paper construes all the available guidance cited above as ‘decision guides’ for researchers and Research Ethics Committees (RECs) on how to obtain valid consent. The paper starts with remarks on the general relationship between ‘decision-guides’, and actual decision-making. The gist of these remarks is that in practice, such pre-determined ‘decision guides’ are *not always* all there is for robust ethical decision-making. Next, while analyzing the five conceptual tools, the paper illustrates that it is this type of relationship (between ‘decision guides’ and ‘actual decision-making’) that accounts for the persistence of seeming inadequacies of the official ethical guidelines (such as the CIOMS guidelines) and other kinds of pre-determined guidance. Finally, the paper presents the sixth concept as a tool by which to appreciate the various ways of filling the gaps between ‘pre-determined guidance on how to obtain valid consent’ on the one hand, and ‘actual decision-making on how consent will actually be obtained in concrete situations’, on the other.

## Main body

### Rules, principles, guidelines, and decision-making

As a background against which to appreciate the conceptual tools proposed in this paper, it is imperative to provide some remarks about the general relationship between rules, principles, and guidelines as ‘decision-guides’ on the one hand, and how they generally relate to actual decision-making. The contention underlying these remarks is that generally, rules, for example, legal rules, are general decision-guides to judicial decision-making in an analogous manner that ethical principles, guidelines and ‘prior conceptual and empirical research’^1^ are to bioethical decision-making. As has been suggested elsewhere, these insights can inform the way we ought to construe the relationship between ethical principles and guidelines in bioethical decision-making [37]. The appreciation of this relationship provides initial insight into *why* existing guidelines many times *appear inadequate* in the process of obtaining and ascertaining valid consent.

Drawing on the philosophy of judicial decision-making, a recent account of ‘bioethical realism’ as a framework for implementing universal research ethics, has used an analogy of ‘legal realism’ to demonstrate that in bioethical decision-making, ethical principles and guidelines are “not always all there is” in concrete situations [37]. Just as legal rules are said to be too general and indeterminate to always provide straight-forward answers in settling specific cases [38], ethical guidelines and other varieties of pre-determined guidance suffer from the same fate in bioethical decision-making. In the case of the juridical analogy, the point is that during legislation it is not possible to foresee all the relevant facts of future cases to which legal rules will apply, in order to be able to provide specific guidance on such cases. To emphasize this point, legal realists observe that even in relatively homogeneous and static societies, “[ …] men have never been able to construct a comprehensive, eternized set of rules anticipating all possible legal disputes and settling them in advance. [ …]. No one can foresee all the future permutations and combinations of events; situations are bound to occur which were never contemplated when the original rules were made” [39]. It is this human epistemic fallibility phenomenon, more so of the knowledge of the future, that explains why it is not always possible for robust decision-making to exclusively rely on predetermined criteria.

Following from the above, in a similar way, while ethical principles and guidelines plus additional evidence from research as ‘decision-guides’ provide invaluable guidance towards actual decision-making (on how to obtain valid consent), there will always remain unique and unpredictable cases in which decision-making will need to be supplemented by, or rely on something more than official ethical guidelines and additional guidance from prior research. So, in an effort to close the gaps left by ethical guidelines, in addition to such prior conceptual and empirical research, these gaps can be closed by an attitude and skill called *bioethical reflexivity* as demonstrated later in this paper. As will be seen later, this concept refers to the willingness and ability to critically reflect and evaluate concrete situations and attempt to make the most morally appropriate decisions in those specific circumstances, as opposed to always relying entirely on pre-determined criteria which sometimes leads to perceptions of inadequacy.

As demonstrated below, given the wide variations in factors that validate consent,^2^ and given the impossibility of foreseeing which of those will hold true in different studies and contexts (concrete situations), the process of ethical guideline-making is often characterized by uncertainties. Even though these uncertainties are currently minimized by prior conceptual and empirical research in local contexts, some level of uncertainty persists with regard to the relevant variables of future studies, including future circumstances of the community in which studies will be conducted and individual traits of potential study participants. This is what is called *fact-skepticism* in this paper. As analyzed below, the concept of *fact-skepticism* is intended to convey a state of ‘uncertainty’ that pervades the process of ethical guideline-making and also left by prior research. Consequently, it becomes necessary to frame the guidelines in a *general*, *context-neutral* and *presumptive* manner. But in turn, due to these three features, it means that it is *not always* possible for the guidelines to provide straight-forward answers to specific and unique questions as they arise in concrete situations. This is what explains the *perception of guideline-inadequacy* in actual decision-making. However, it will further be shown that this is not a flaw of the guidelines but their inherent mechanism that allows their effective application in various situations.

### On obtaining and ascertaining ‘valid consent’

On the basis of the above discussion, the framing of the problem at hand suggests that obtaining and ascertaining valid consent in research does *not entirely* rely on an application of preexisting knowledge, whether in the form of existing ethical guidelines or findings from prior research. Instead, it suggests a holistic approach to consent in which, on top of taking ethical guidelines as starting points, obtaining valid consent is more of an evaluative process, partly based on the discretion of researchers and RECs in consideration of specific and unique features of each context. This explains the spirit of, for example, the CIOMS guidelines’ provision for “Waivers and modifications of informed consent” (guideline 10), the importance of ‘Community Engagement’ (guideline 7) [3], as well as what has been described as ‘Rapid Assessments’ [1, 40]. By being an evaluative process, it means that some of the specific criteria for obtaining valid consent as set out in different guidelines may sometimes be found inappropriate or insufficient to ensure ‘valid consent’ in some contexts. The conceptual analysis and arguments which follow below are intended to provide: 1) a conceptual background against which to understand the unavoidable gaps between pre-determined guidance for decision-making and actual decision-making and 2) to appreciate the importance of practices such as ‘Community Engagement’ and ‘Rapid Assessments’ which are examples of reflexivity in health-related research.

### Conceptual resources

#### Fact-skepticism

The point to be demonstrated by the concept of *fact-skepticism* is that the persistence of the seeming inadequacies in existing ethical guidelines as decision-guides on how to obtain valid consent is explained by the uncertainty that pervades the process of making ethical guidelines. This uncertainty is about which, of the factors that are relevant in determining the validity of consent, will hold true in concrete situations. Generally, in cases of decision-making where the process is expected to rely on decision-guides such as ethical principles and guidelines, such decision-guides are usually applied to specific variables to produce specific decisions. In this case, the relevant factors or variables (as analogues of *facts* in a juridical analogy) are, among others, study-related variables such as the type of the study – say, genomic studies or HIV phylogenetic studies – along with their methodological designs and procedures etc.; and, on the other hand, the nature of the community and characteristics of target individual participants. On the other hand, the concept of *skepticism* is intended to convey a state of *indeterminacy* or *uncertainty.* From an epistemological discourse generally, skepticism is a theory that certain (indubitable/perfect) knowledge is impossible [41], and much so, knowledge of the future. Following from the discussion above, uncertainty applies to the specific variables on which decision-guides will apply.

Consequently, the concept of *fact-skepticism* in research ethics is based on the contention that existing ethical principles and guidelines and additional guidance from prior research are not always based on actual scenarios in which decisions will actually be made, but on those that can be *reasonably* predicted, presumed or imagined. But given human epistemic limitations, especially knowledge of the future, many times the actual scenarios in the field tend to deviate from, and/or supersede those which were presumed by framers of ethical guidelines as well as guidance provided by prior conceptual and empirical research on the same subject. The practical implication of *fact-skepticism* is that in the application of existing ethical guidelines, researchers and RECs should be always willing and able to judiciously fill the gaps that usually emerge between pre-determined guidance on how valid consent *ought* to be obtained generally, and how valid consent *will* be obtained in a specific study.

#### ‘Guideline-inadequacy’ as an analogue of ‘rule-skepticism’

Using the analogy of the time-tested concept of *rule-skepticism* in the discourse on judicial decision-making, the concept of *guideline-inadequacy* is intended to further demonstrate why officially-specified criteria in existing ethical guidelines and all sorts of pre-determined guidance on how to obtain valid consent are *not always* all there is in making such decisions in concrete situations. According to Wilfrid E. Rumble, in the juridical discourse, the notion of ‘*rule-skepticism*’ is a corollary of *fact-skepticism* [39]. That is, our inability to foretell with prophetic certitude what the actual facts will be in specific legal disputes, means that the laws, as judicial decision-guides, *cannot always* provide precise and straight-forward answers to all disputes. Generally, in the juridical discourse, the concept of *rule-skepticism* is intended as a caution about the insufficiency of pre-determined general legal rules (including judicial precedents) in settling specific legal disputes. Drawing insights from Karl R. Llewellyn’s views on the reality of judicial decision-making [42], Rumble defines the concept of ‘*rule-skepticism*’ as follows: “By this is meant the theory that established rules have not been, *in most cases*, the decisive factors determining judicial decisions” [39] (my emphasis). In an attempt to reveal the unavoidable phenomenon of rule-insufficiency in judicial decision-making, for example, all legal realists agree that in a majority of instances, ‘paper rules’ (pre-determined legal rules) are not all there is in judicial decision-making [39, 42–44]. This is because “general propositions do not determine concrete cases [ …]. [Consequently], until general rules are interpreted and applied to specific concrete cases, their actual meaning for, and bearing upon concrete situations cannot be fully ascertained” [39]. For the same reason, despite existing guidance on how to obtain valid consent, valid consent remains both a normative and practical challenge [45] *a fortiori,* if we fall into the temptation of believing that an existence of pre-determined ethical guidance whether from official ethical guidelines or additional evidence from prior research are sufficient for robust consenting processes that can be reasonably expected to yield valid consent.

However, for clarity and emphasis, it needs to be noted that the concept of guideline-inadequacy does not entail that existing ethical guidelines and additional guidance from prior research are unimportant. It only means that a set of pre-determined criteria for decision-making is *not always* all there is for robust bioethical decision-making processes. This contention is as true for obtaining and ascertaining valid consent for participation in research, as it is for judicial decision-making. Hence, in addition to such general decision-guides including evidence from empirical studies, certain skills and attitudes are to be expected in order to prudently fill the gaps between existing guidelines and actual practice.

### The ‘generality’, ‘context-neutrality’ and ‘presumptiveness’ of ethical guidelines

The upshots of the two concepts discussed above are three closely related concepts that explain the essential nature of existing ethical guidelines, the very nature that further accounts for perceptions of *guideline-inadequacy,* despite substantial additional guidance provided by findings from more local empirical research on the same subject. These concepts are *Generality*, *Context-neutrality,* and *Presumptiveness*.

#### The ‘generality’ of the guidelines

To be general means that the guidelines are concerned with expounding on universal ethical principles and, bearing in mind the variations in specific contexts in which they are meant to apply, suggesting how, *all things being equal*, such principles ought to be applied in decision-making. So, as opposed to providing a set of rigid and exhaustive criteria for decision-making, the guidelines simply state basic and general considerations. The generality of ethical guidelines is dictated by the pervasiveness of *fact-skepticism* at the point of guideline-making and at the same time this generality leads to perceptions of *guideline-inadequacy*. The inevitability of the generality of ethical guidelines (whether international, regional or national) can be demonstrated with George Soros’ views on the human capacity to comprehend reality and the consequence of human epistemic limitations. As will be expounded on later, Soros argues that the extreme complexity of reality means that we cannot gain full knowledge of how reality works, and as a result we usually “resort to various methods of simplification such as decision rules, moral precepts, *generalizations*, dichotomies, and metaphors” [46] (emphasis added).

The contention that existing ethical principles and guidelines are simply *general* starting points into bioethical decision-making can be demonstrated using excerpts from the CIOMS guidelines, particularly on consent. Whereas some of the questions that need to be answered in the process of obtaining valid consent have to do with, among others, the type and amount of information that ought to be given to study participants [14, 15] and what degree of comprehension is sufficient for the resulting consent to be valid [21–24], all the guidelines say is that researchers should provide the “*relevant information”* about the research and ascertain that potential participants have “*adequate understanding of the material facts*” and also give participants “*sufficient opportunity and time”* to decide whether they want to participate (Guideline 9), [3] (emphasis added). Further, even though CIOMS Guideline 9 partly states that “as a general rule” researchers ought to obtain *written evidence* of consent from participants, it still acknowledges the possibility that this will not always be the case, although the guideline asks researchers to justify whatever exceptions they make to this requirement [3]. Further, CIOMS guideline 10 adds to the evidence of the generality of the guidelines: it allows discretion to researchers, in consultation with, and approval by the relevant RECs, to modify and waive requirements of informed consent, including waiving consent even where study risks may be slightly more than minimal [3]. The practical significance of appreciating this deliberate generality is that it is up to researchers, with the approval of RECs, to decide and justify, for example, which and how much information about a study is *relevant and sufficient*; precisely define levels of understanding/comprehension that are adequate (and how such levels of comprehension will be measured/ascertained), and how much time and opportunity are sufficient for different potential participants to make free and informed decisions on whether to participate in a study. Consequently, since the guidelines do not dictate specific answers to these questions, this deliberate *generality* further explains why existing guidelines sometimes seem to provide insufficient guidance on how to obtain valid consent. By implication, the guidelines are designed in such a manner that researchers and RECs exercise reflexivity by way of always being ready and able to take on the responsibility of making and accounting for specific decisions in concrete situations.

#### Context-neutrality

Related to the *generality* of ethical guidelines, is their *context-neutrality*. The point of context-neutrality is that since we can never be absolutely sure about all specific contexts and concrete circumstances (variables) in which ethical guidelines will ultimately be applied, it becomes not just important but also *necessary* for the guidelines to be stated in a context-neutral manner to allow the exercise of discretion in choosing the most appropriate way to obtain consent in different contexts. Hence, even though the guidelines try as much as possible to specify instances to which the guidelines are expected to apply, ultimately the examples cited (from CIOMS guidelines) above indicate that existing ethical guidelines are intended to be largely context-neutral. A further example from CIOMS guidelines is that on top of specifying provisions for possible waivers and modifications of informed consent, the guidelines add that “Additional provisions may apply when waivers or modifications of informed consent are *approved in specific research contexts”* [3] (emphasis added). In addition, still in reference to the issue of consent in the CIOMS guidelines, guideline 7 can be used to demonstrate the implicit suggestion of context-neutrality. According to the CIOMS guideline 7 on “Community Engagement”, one of the goals of ‘Community Engagement’ is to enable communities in which studies are taking place to have input into, among others, the design of the informed consent process [3]. Consequently, while the guidelines provide initial and general criteria for making decisions on how to obtain valid consent, ultimately such decisions are context-specific, to the extent that we should not be so worried about the seeming failure of the guidelines to provide decisive answers on how to obtain valid consent. Rather we should ask what conceptual and practical tools that can enable us to navigate the seeming inadequacy of the guidelines in bioethical decision-making. One tool of such kind defended in this paper is *bioethical reflexivity* which makes intelligible the essence of some of the practices such as ‘Community Engagement’ and ‘Rapid Assessments’ in health-related research.

#### The ‘presumptiveness’ of ethical guidelines

Another concept that can be used to explain the nature of ethical guidelines is ‘presumptiveness’. In their discussion of how the four principles of bioethics (justice, non-maleficence, beneficence, and autonomy) ought to be understood and applied, Beauchamp and Childress indicate that among other things, the principles are presumptive by nature [47]. This means that such principles *presume* certain truths about situations in which they will be applied while assuming ‘other factors constant’. Hence, to say that ethical guidelines are ‘presumptive’ by nature is to say that they are stated with a silent proviso – ‘all things being equal’. For clarity, in this case, ‘things’ should be understood as a number of study-related variables as well as the characteristics of communities and individuals among whom the guidelines will be applied. This proviso implies that if the variables in concrete situations were to be found exactly those presumed in the guidelines, then decisions ought to be made exactly as stated in the guidelines, including obtaining valid consent. But as matter of fact, other factors are not always constant and in concrete situations, there are usually more specific factors that differ from, and/or supersede those cited or presumed in the guidelines, even those that inform prior research on the same subject. In other words, even where the guidelines provide elaborate lists of instances and what ought to be done therein, they implicitly acknowledge that ‘all things are not always equal’. With the help of a hypothetical illustration, we can better appreciate the presumptive character of the guidelines.

The contention here is that guidelines can, for the *most* part, to say the least, be understood as saying that ‘all things being equal, if you were to conduct a study of nature ‘N’ involving procedures ‘P_1_, P_2_, P_3_, … P_n_’; in a community with socio-economic traits ‘T_1_, T_2_, T_3_, … T_n_, and individual participants with qualities Q_1_, Q_2_ Q_3_ … Q_n_, etc., then the right things to do in order to obtain valid consent are ‘X, Y, Z’. Hence, the presumptive nature of the guidelines can be better appreciated against the backdrop of *fact-skepticism* that pervades ethical guideline-making and traces of skepticism left by prior research. Further, this nature explains why *bioethical reflexivity* as currently operationalized in the form of related practices such as ‘Community Engagement’ and ‘Rapid Assessments’, is a handy conceptual and practical tool in obtaining valid consent to research participation.

### The guidelines’ internal mechanism against perceived inadequacy

Generally, the concepts suggested above as tools by which to better understand the nature of existing ethical guidelines are at the same time intended to make intelligible why, despite the existence of these guidelines along with the volume of empirical research conducted on how to obtain valid consent, in practice, the process remains a challenge. The conclusion which can be drawn from the above concepts is that in the process of obtaining consent, there will always be unprecedented and unforeseen scenarios of ethical significance on which pre-determined ethical guidance alone, howsoever detailed and localized, will be inadequate. However, the guidelines’ nature as analyzed above which reveals their seemingly inevitable inadequacy, is not their weakness but their strength. This nature – generality, context-neutrality, and presumptiveness – is their *internal mechanism* for enabling the making of ethically appropriate decisions in unforeseen scenarios that sometimes arise in concrete situations. This mechanism comes in the form of the room for discretion and responsibility the guidelines assign to agents responsible for making final decisions on how consent ought to be obtained in concrete situations. But in order to emphasize the critical importance of the concept and practice of bioethical reflexivity as analyzed below, it is important to underscore the view that such an internal mechanism simply presents a *potential* for overcoming seeming inadequacies. Transforming such potential to practicality depends on the agency of those making decisions; that is, the willingness and ability of researchers and RECs to effectively transform such potential into reality. The concept that conveys the manner in, and skill and attitude with which researchers and RECs can effectively close the gap between these guidelines and actual practice, is the concept and practice of *bioethical reflexivity*.

#### Bioethical reflexivity

The use of the concept ‘reflexivity’ in research, including research ethics, is not an invention of this paper. It has been claimed that this conceptual and practical tool ensures rigor in decision-making while responding to unique moral issues as they arise in the conduct of research [48–52]. Further, as mentioned above, there are some current practices in research, including processes of obtaining consent that might reflect what reflexivity partly entails. Some of these practices include ‘Community Engagement’ and what has been termed ‘Rapid Assessments [40]’. The latter has been demonstrated as a relatively quick and inexpensive way of tailoring the provision of study information and the whole consent process to contexts [1]. However, this paper provides a conceptual analysis of the guidelines’ nature as a critical background against which the essence of reflexivity as partly operationalized in these and similar practices can be best appreciated in an attempt to close the gaps left by existing ethical guidelines, particularly on how to obtain valid consent.

### Meaning of ‘reflexivity’

In an effort to analyze the meaning of the concept of bioethical reflexivity, this paper first points at the fundamental connotations of the concept of ‘reflexivity’ and later applies it to the concept of bioethics to come up with one compound concept. Looking at the numerous discourses in which the concept of ‘reflexivity’ is broadly used [53] helps identify its fundamental connotations. These usages emphasize a habit of constant critical appraisal of one’s intentions and *means*, in light of certain goals as opposed to exclusive reliance on traditional or pre-determined ways of doing things. In postmodern philosophy, the concept of ‘reflexivity’ (not necessarily the term) can be partly attributed to some of Karl R. Popper’s works: one, *The Open Society and Its Enemies* [54] and two, *The Logic of Scientific Discovery* [55]. The aim of these two works is the same – to demonstrate human epistemic limitations in relation to the truth about social and scientific realities, respectively. From Popper’s analysis, George Soros derives the “Human Uncertainty Principle” that necessitates both the concept and practice of “reflexivity” [46]. The major contention underlying Popper’s views in the two works cited above is that empirical truth, including social and scientific/empirical reality or truth, cannot be verified beyond a shadow of a doubt. It is for this reason that he demonstrates that even “Scientific laws are always hypothetical in character, and their validity remains open to falsification” [46].

It is the above phenomenon (human epistemic fallibility) that has been termed ‘fact-skepticism’ in this paper. As a solution, Soros argues, when confronted with this sort of extreme complexity, we end up resorting to various methods of simplification such as decision rules, moral precepts, generalizations, dichotomies, metaphors among others [46]. Consequently, truth about reality remains open-ended, and one of the contentions of this paper is that this open-endedness equally applies to the various variables which usually play a role in validating consent to research participation in concrete situations*.* Hence, since actual decisions on how to obtain valid consent in concrete situations are themselves ‘close-ended’ in nature (decisions on what will *actually* be done as opposed to what *might* or *could* be done), in actual decision-making there needs to be something more than open-ended decision-guides including official ethical guidelines and the various prior views generated from both conceptual and empirical research trying to answer the same question. Although these pieces of knowledge from empirical research somehow reduce the depth of skepticism, given the inevitability of human epistemic fallibility, some level of skepticism persists. Consequently, in actual decision-making there is usually a need for something more than pre-determined guidance on how to obtain valid consent – reflexivity. In this case reflexivity ought to be understood as the *willingness* and *ability* to constantly and critically reflect on the existing decision-guides with a view of identifying which ones are appropriate or inappropriate in concrete situations, including *how*, and *why,* and then going further to judiciously decide what, in the circumstances, the most appropriate thing to do is.

### Reflexivity in research ethics

While discussing the concept of reflexivity in research ethics, some have contended that “ethical research is much more than research that has gained the approval of RECs” [50], especially so when difficult and unexpected situations arise in the field and researchers are forced to make immediate decisions about ethical concerns. For emphasis, these viewpoints point at the inevitable inadequacies of pre-determine ethical criteria as decision guides for prudent bioethical decision-making generally. In the view of these authors, it is in situations of this sort that reflexivity becomes an important tool. The emphasis is that the alertness suggested by the practice of reflexivity might include conscious considerations of a range of formal ethical positions and the adoption of a particular ethical stance. Hence, reflexivity encourages researchers to develop skills needed to enable morally appropriate responses to unique and unprecedented events of ethical significance, including those incapable of being resolved by reference to existing guidelines and/or additional guidance from both conceptual and empirical research. As a result, “a reflexive researcher will be better placed to be aware of *ethically important moments* as they arise and will have a basis for responding in a way that is likely to be ethically appropriate, even with unforeseen situations” [50].

Therefore, as implicitly suggested in various official guidelines, in order to make morally appropriate decisions on the process of consent in concrete situations, researchers and RECs have a responsibility of prudently filling the gaps left by the guidelines. Further, since the process of obtaining valid consent is an evaluative one as opposed to being a strictly logical undertaking, it means that on top of acquiring sufficient knowledge of the guidelines, and acquainting oneself with as much guidance as possible from prior conceptual and empirical research, there is need to emphasize the importance of reflexive mindsets and skills in both abstract and practical moral reasoning. For similar reasons, a number of views in methodologies of bioethics have repeatedly called for the development of critical thinking in bioethics [56–59]. In closing remarks, it is important to say something more about potential controversy arising from the discretion suggested by the concept and practice of *bioethical reflexivity*.

### Bioethical reflexivity and ethics accountability in research

If the concept and practice of bioethical reflexivity were to be restricted to practices such as ‘Community Engagement’ and ‘Rapid Assessments’ per se, then the following controversy would not have potential. But the concept demands much more than these practices in and of themselves. The aptitudes and skills suggested by the concept of ‘reflexivity’ suggest significant use of researchers’ discretion even while conducting ‘Community Engagement’, ‘Rapid Assessments’ and other potential practices for a similar purpose. Consequently, there may arise worry about potentially negative implications of the concept and practice of *bioethical reflexivity* due to the discretion it suggests for researchers and RECs. Generally, the reason for specifying ethical principles and guidelines for research and how they could be implemented is a realization that researchers *cannot always* effectively regulate themselves. That is to say, their discretion and goodwill cannot always be relied upon in deciding what is morally appropriate in research involving human participants. For this reason, ethics accountability is critical in research, especially so when some of the studies’ ethical designs turn out to be controversial, yet they were approved by RECs. The possibility of robust ethical accountability in research presupposes the existence of pre-determined and objective criteria in the form of standard principles and guidelines that should be referred to in the process. However, the concept of bioethical reflexivity with its consequent discretion on the part of researchers and RECs may seem to open floodgates for arbitrariness, making robust accountability difficult.

On the contrary, however, the discretion suggested by the concept and practice of *bioethical reflexivity* does not mean that we must always sanctify whatever decisions are made between researchers and RECs, at all cost. This further explains the essence of ‘Community Engagement’ and ‘Rapid Assessments’ mentioned earlier. Without such practices as manifestations of reflexivity, it is possible to imagine cases where researchers and RECs agree on certain decisions regarding the ethical design of a study, only for those decisions to turn out to be controversial in the opinion of other relevant stakeholders such as the general public. As matter of precedence, not necessarily for consent-related reasons, some civil society organizations have successfully challenged the ethics of certain studies which had been duly approved by RECs, leading to early termination of such studies in a number of countries, as cited by Bhan Anant and colleagues in reference to HIV/AIDS pre-exposure prophylaxis trials [60]. It is possible that similar agitation can arise for reasons related to the quality of consent processes. In such instances the burden of proof is borne by the researchers and sometimes together with RECs, to demonstrate to other relevant stakeholders including the public, that the decisions they made and actions taken, were the most ethically appropriate in the circumstances. Further, since ‘Community Engagement’ is not just a formality but must be *meaningful*, according to the CIOMS guidelines, achieving this meaningfulness will equally depend on the discretion of the researchers on how ‘Community Engagement’ and ‘Rapid Assessments’ will be conducted, including their timing, who gets involved, what aspects of the study *need* to be discussed in the process, what information will be revealed, among others. This further means that since researchers must exercise their discretion in the conduct of ‘Community Engagement’, it is possible for the public to question the meaningfulness of these processes. It is these various ways of holding researchers and RECs accountable for their decisions that significantly limit potential widespread abuse of the discretion suggested by the concept and practice of bioethical reflexivity.

## Conclusions

The problem with existing ethical guidelines for health-related research is not that in many cases they fail to provide sufficient guidance as to how exactly specific and unique questions in the field should be answered. By their very nature, they ought not to be expected to always do so, howsoever detailed and localized they may be. Further, even though more localized empirical research usually makes up for some of the uncertainties that characterize processes of making these guidelines, given the pervasiveness of human epistemic limitations, such studies cannot always fully close the gaps between guidelines and concrete decision-making scenarios. The unknowability of all the relevant variables of future studies, as well as the inability of prior empirical and conceptual research to fully close all the gaps, necessitates that guidelines be stated in a more general, context-neutral and presumptive manner to allow those making final decisions exercise discretion in responding to unique scenarios in the field. So, what is usually perceived as the guidelines’ inadequacy is their internal mechanism-cum-potential to facilitate their effective application in highly diverse and, usually, unpredictable contexts. Taking full advantage of this potential requires certain attitudes and skills on the part of researchers and RECs, and these attitudes and skills can be best conveyed by the concept and practice of reflexivity, hence, *bioethical reflexivity*.

## Data Availability

Not applicable.

## Abbreviations

CIOMS: Council for International Organization of Medical Sciences
HIV/AIDS: Human Immune Virus/Acquired Immuno-Deficiency Syndrome
RECs: Research Ethics Committees

## Footnotes

1 By ‘prior conceptual and empirical research’ is meant research conducted long before the actual study for which consent is being sought. The view of this paper is that project-specific research such as that described as ‘Rapid Assessment’ to address specific ethical needs of studies is considered to be a practice of reflexivity.

2 The major three factors that determine the validity of consent are: Adequacy of information (which varies between studies); Comprehension of the provided information (which depends on other many variables such as literacy levels, language, method of delivery etc.) and Freedom/voluntariness (which also depends on many variables, such as the nature of the relationship between researchers and research participants (e.g. doctor-patient relationship); different kinds of vulnerabilities e.g. desperate medical needs, economic poverty among others).
